# Molecular Dynamics Simulations to Provide Insights into Epitopes Coupled to the Soluble and Membrane-Bound MHC-II Complexes

**DOI:** 10.1371/journal.pone.0072575

**Published:** 2013-08-19

**Authors:** Martiniano Bello, Jose Correa-Basurto

**Affiliations:** Laboratorio de Modelado Molecular y Bioinformática de la Escuela Superior de Medicina, Instituto Politécnico Nacional, México, Plan de San Luis Y Diaz Mirón S/N, Col. Casco de Santo Tomas, Mexico City, México; Oak Ridge National Laboratory, United States of America

## Abstract

Epitope recognition by major histocompatibility complex II (MHC-II) is essential for the activation of immunological responses to infectious diseases. Several studies have demonstrated that this molecular event takes place in the MHC-II peptide-binding groove constituted by the α and β light chains of the heterodimer. This MHC-II peptide-binding groove has several pockets (P1-P11) involved in peptide recognition and complex stabilization that have been probed through crystallographic experiments and *in silico* calculations. However, most of these theoretical calculations have been performed without taking into consideration the heavy chains, which could generate misleading information about conformational mobility both in water and in the membrane environment. Therefore, in absence of structural information about the difference in the conformational changes between the peptide-free and peptide-bound states (pMHC-II) when the system is soluble in an aqueous environment or non-covalently bound to a cell membrane, as the physiological environment for MHC-II is. In this study, we explored the mechanistic basis of these MHC-II components using molecular dynamics (MD) simulations in which MHC-II was previously co-crystallized with a small epitope (P_7_) or coupled by docking procedures to a large (P_22_) epitope. These MD simulations were performed at 310 K over 100 ns for the water-soluble (MHC-II_w_, MHC-II-P_7w_, and MHC-II-P_22w_) and 150 ns for the membrane-bound species (MHC-II_m_, MHC-II-P_7m_, and MHC-II-P_22m_). Our results reveal that despite the different epitope sizes and MD simulation environments, both peptides are stabilized primarily by residues lining P1, P4, and P6-7, and similar noncovalent intermolecular energies were observed for the soluble and membrane-bound complexes. However, there were remarkably differences in the conformational mobility and intramolecular energies upon complex formation, causing some differences with respect to how the two peptides are stabilized in the peptide-binding groove.

## Introduction

Major histocompatibility complex (MHC) molecules are heterodimeric proteins that bind antigenic peptides as part of the adaptive immune response to foreign pathogens. MHC class I (MHC-I) presents primarily peptides of endogenous origin, whereas MHC class II (MHC-II) binds molecules derived from exogenous proteins. These exogenous molecules are commonly short peptides, the products of the degradation process for exogenous proteins. After a peptide binds to a MHC-II molecule to create a pMHC-II complex, the peptide is presented to T-cell receptors (TCRs), which identify foreign antigens [1]. Unlike MHC-I, whose peptide-binding groove is geometrically optimized to bind small peptides smaller than 11 residues in length, MHC-II has a binding groove that is open at both ends, thus allowing peptides of varying lengths (12 to 26) to bind [2]–[4]. Only a core of nine consecutive residues interacts with the MHC-II molecule at certain anchor residues [5]. Furthermore, this peptide core can be flanked by a variable number of residues [4], which could enhance the processing of epitopes and modulate the activation of T cells after reaching the TCR [6].

X-ray studies have provided some evidence of the rules that govern peptide recognition by MHC-II. First, despite the lack of significant structural variation among the crystallographic structures of peptide-free MHC-II and pMHC-II forms, alternate conformations have been reported for both MHC-II states [7]–[12]. In fact, an increase in the hydrodynamic radius and a decrease in helicity have been observed for the peptide-free form of MHC-II (DRB1*0101) with respect to pMHC-II [12], [13]. Interestingly, the opposite behavior occurs upon peptide binding, suggesting that there is higher conformational mobility for the peptide-free MHC-II form. Second, the peptide in the peptide-binding groove adopts a type II polyproline helix, which causes the peptide to twist in a specific manner, with the sequestration of the peptide side chains in the polymorphic pockets (Ps) of the MHC-II molecule [14], [15]. Generally, these Ps accommodate the side chains of peptide residues and can be divided into two classes: the class comprising P1, P4, P6, and P9, which have been identified as major anchors and are localized in solvent-inaccessible regions, and the class comprising P2, P3, P7, and P10, which are smaller pockets that function as auxiliary anchors [16], [17]. This type of molecular recognition has been interpreted as a docking event stabilized by a series of sequential and independent interactions formed between residues of the peptide and Ps [18].

With respect to the conformational behavior of MHCs in aqueous environments, most MD simulations performed with MHC molecules have been focused on MHC-I [19]–[21]. Based on these studies, we know that MHC-I experiences a reduction in conformational mobility upon pMHC-I complex formation [22]; however, such studies took into consideration only the peptide-binding groove (chains α and β) and not the whole MHC-I molecule. Later, Wan et al. showed that MD simulations that do not take into account the entire complex (light and heavy α and β chains) could give misleading results for the conformational mobility and the estimated binding free energy [23]. Knapp et al. explored the binding between MHC-II and two peptides with different flanking regions but the same epitope core, demonstrating that the larger peptide has greater flexibility in the peptide-binding groove of MHC-II and a higher binding affinity than the smaller peptide [24]. In addition, MD simulations have been used to study a TCR-pMHC-II-CD4 complex anchored in a lipid membrane [25], but that study was more focused on evaluating the free energy values of the binding between the TCR and MHC-II than those of the pMHC-II complex, which were estimated without taking into account the entropy component. Furthermore, Wan et al. did not investigate pMHC-II or peptide-free MHC-II in aqueous solution to allow comparisons with the systems simulated in a membrane environment [25]. Therefore, in our opinion there has been no previous study that investigated the importance of the conformational stability of MHC-II in a membrane environment. One may ask whether the lack of anchoring to a membrane can be neglected when simulating a pMHC-II complex, as reported by our research group [26].

To answer this question, in this study, we explored the conformational and energetic changes of water-soluble peptide-free MHC-II (MHC-II_w_) and MHC-II_w_ coupled to a small (P_7_) or a large peptide (P_22_), denoted MHC-II-P_7w_ and MHC-II-P_22w_, respectively. These MHC-II complexes were also anchored to a POPC membrane through parts of theirs α and β heavy chains, referred to as MHC-II_m_, MHC-II-P_7m_ and MHC-II-P_22m_, respectively.

These MD simulations showed that the peptides in the MHC-II-P_7,22_ complexes in either the aqueous or membrane environment interacted with P1, P4, P6-7. However, despite having similar noncovalent intermolecular energies, these complexes exhibited differences in their conformational fluctuations and in their intramolecular energies upon complex formation, revealing that the whole system is more stable than the water-soluble domain alone.

## Methods

### MHC-II_w_, MHC-II-P_7w_ and MHC-II-P_22w_ models

**Figure 1:**
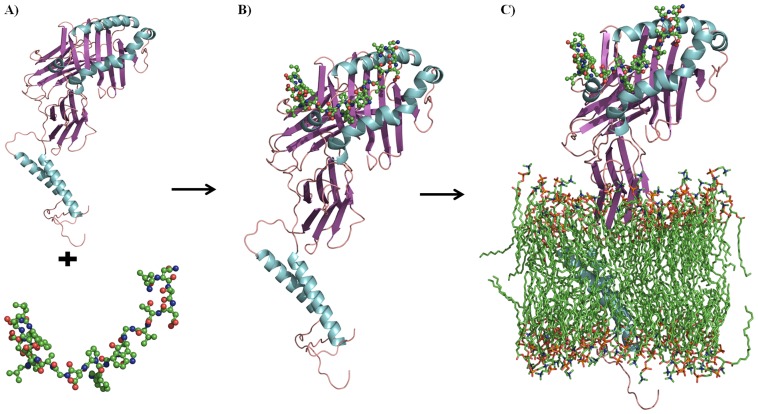
Steps depicting the construction of the membrane-bound systems (MHC-II_m_, MHC-II-P_7m_ and MHC-II-P_22m_). A) The receptor (MHC-II) and the ligand (P_22_). B) The MHC-II-P_22_ complex. C) The MHC-II-P_22m_ complex embedded in a POPC membrane.

The MHC-II-P_7w_, MHC-II-P_22w_ and MHC-II_w_ models were based on the co-crystallized pMHC-II complex (HLA-DRB1*0401, PDB entry 1D5M). 1D5M consists of an MHC-II heterodimer co-crystallized with a peptide mimetic inhibitor and a SEB molecule. This peptide mimetic inhibitor contains chemically modified amino acid residues that were modified in this work to maintain the natural residues (ARAMCSL, P_7_). In addition, the three-dimensional (3D) structure of P_22_ (VNSDTVGWSWPDGAELPFTIDK) was built using the I-Tasser server [27], and the structure with the highest C-score was selected. The MHC-II-P_22w_ model was constructed using the apo form of MHC-II (1D5M) and a docking procedure to couple MHC-II to P_22_, which is a neuraminidase peptide [26]. The docking study was performed using the Cluspro 2.0 server [28], [29]. This rigid-body protein docking program was chosen because has been one of the top performers at CAPRI (Critical Assessment of Predicted Interactions) rounds 1–12, the community-wide experiment devoted to protein-protein docking [30]. And among all the models generated by Cluspro, we select one of the returned models with the lowest energy, the highest score values (fig. 1A and B) and that in which P_22_ was stabilized by residues in Ps P1, P4, P6-7, since it has widely stated that interactions between these Ps and MHC-II are essential in this class of molecular recognition [16]–[18]. The free MHC-II model was created by deleting the peptide mimetic inhibitor and the SEB molecule from the co-crystallized complex (1D5M). The missing loops were constructed using CPH-models [31].

### MHC-II_m_, MHC-II-P_7m_ and MHC-II-P_22m_ models

The procedure used to model the membrane-bound system was the following: because structural information for the MHC-II molecule is only available for the soluble region (PDB entry 1D5M), molecular modeling was performed to construct the transmembrane and cytoplasmic region. The amino acid sequences of HLA-DRB1*0401 (P01903 and P13760) were retrieved from NCBI. The template selection was done for each of the targeted sequences (PDB entry 1D5M), first 1D5M (α-domain) was alignment to sequence P01903 and 1D5M (β-domain) to P13760 using BLAST [32]. Later on, the model building was carried out using MODELLER Version 9.10 [33].

The orientation of free (MHC-II_m_) and the complex forms (MHC-II-P_7m_ and MHC-II-P_22m_) in the membrane was predicted using OPM (Orientations of Proteins in Membranes) server [34] which was consistent with experiments [35]–[38]. Based on this information, the free and the complex forms were inserted into a pre-equilibrated palmitoyl oleoyl phosphatidylcholine (POPC) bilayer consisting of 128 lipid molecules (D. P. Tieleman's site, http://moose.bio.ucalgary.ca). First, these structures were symmetrically oriented according to the X-Y-Z box vectors of the POPC unit cell using editconf such that the MHC-II components were solvent expose, whereas the complex was anchored to POPC membrane through two α-helices, which form part of its α and β heavy chain (fig. 1C). Second, pMHC-II_m_ were inserted into the POPC membrane using the g_membed method [39] and minimized for approximately 2 ns. Before solvating and neutralizing the system, the z-axis of the membrane bilayer was increased so that it was sufficiently long to cover the entire MHC-II heterodimer.

MD simulations were conducted using the GROMACS 4.5.3 [40], [41] package with the GROMOS96 53A6 force field [42]. All the systems were energy-minimized before MD, using ∼10000 steps of the steepest descent method, to relax any steric conflicts generated during setup. Histidine residues were protonated on both protonatable amine groups (ND1 and NE2), whereas the rest of the ionizable residues were in their default ionization state. All the systems were solvated with SPC (simple point charge) waters [43], and system-neutralizing sodium and chloride ions (corresponding to ∼0.1 mM NaCl) were added. After energy minimization, the systems were submitted to a 2-ns equilibration period restraining the whole protein and the cation positions. Then, the whole system, including the proteins and lipids, was submitted to unrestrained MD simulations lasting 2 ns using the NVT ensemble and 2 ns using the NPT ensemble. Electrostatic interactions were calculated using the particle mesh Ewald method [44] with a 1.2-nm cutoff for the real-space calculation. A cutoff of 1.2 nm was used for van der Waals interactions. MD simulations lasting 100 (for the soluble systems) and 150 ns (for the membrane-bound systems) were performed for each system at constant temperature, pressure, and number of particles. The temperatures of the protein, POPC, and solvent were each coupled separately using the Berendsen thermostat [45] at 310 K, which is well above the phase transition temperature of pure POPC (271) [46], for the in-solution and POPC simulations, with a coupling constant of *τ*_T_ = 0.1 ps. The pressure was coupled using the Berendsen algorithm at 1 bar with the coupling constant *τ*_P_ = 1 ps. The isothermal compressibility was set to 4.5 × 10^5^ bar^−1^ in all box dimensions. The time step for integration was 2 fs, and coordinates and velocities were saved every 2 ps. The LINCS algorithm was used to restrain the bond lengths [47]. The lipid parameters were based on those used elsewhere [48], [49].

To obtain the projected area per lipid, we divided the area of our simulation box (Box-X times Box-Y from g_energy) by half the number of lipids in our system as determined elsewhere [39]. Intra and intermolecular energies were obtained by calculating the short Lennard-Jones (LJ-SR) and Coulomb (Coul-SR) energies with the g_energy module. The average conformations for the last 85 (for the soluble systems) and 100 ns (for the membrane-bound systems) of the simulations were used to calculate the interaction map between P_7_ or P_22_ and MHC-II in the aqueous and membrane-bound environments. Hydrogen bonds were calculated for the average structures using a donor-acceptor atom cutoff distance of 0.35 nm, and hydrophobic contacts were estimated using an apolar-to-apolar atom pair distance cutoff of 0.5 nm. The GROMACS tools package was used for the data analysis [40], [41]. Images were generated using LIGPLOT v.4.5.3 [50], and structural representations were prepared using PyMOL v0.99 [51].

### Results and Discussion

Before it can exert its biological functions, such as coupling to TCRs, MHC-II has to undergo several oligomer states. After MHC-II biosynthesis, the MHC-II subunits associate with Ii-trimers [52], [53]. Ii-trimers consist of several distinct segments: an N-terminal cytosolic tail [54], a single transmembrane helix, a trimerization domain located at the luminal site of the protein and a short segment termed CLIP that associates with the MHC-II peptide-binding groove and prevents the premature binding of antigenic peptides [55]. Ii-trimers play different roles in the assembly and cellular localization of MHC-II. Nevertheless, although the molecular mechanisms of the association between MHC-II and Ii-trimers have been extensively studied over the past several decades, the structural behavior under physiological conditions remains unclear [56]. Prior to stable MHC-II insertion in the plasma membrane, the complex MHC-II-Ii-trimmer is cleaved through the action of proteases such cathepsin S, leaving CLIP bound to the peptide binding grove, which is later removed by inducing peptide exchange [18]. At the time of maturity, MHC molecules are anchored through their lower part in the cell membrane [35]–[38], where they display short polypeptides to T cells, via the TCRs [57].

As the understanding of the forces that guide the stabilization of a pMHC-II complex is important to gain insight about the peptides with the highest affinity, and therefore those which will be recognized by TCR and awakening an immunological response. Several theoretical studies have been performed to improve our knowledge about this type of interactions. From all these studies, most of them have been MD simulations over MHC-I [19]–[21] and some truncated pMHC-I complexes [22], however, these systems were modeled as soluble systems when it is widely known that MCH-II is not free in solution when it binds to the peptide, but also that is anchored to a cell membrane through part of its α and β heavy chains [35]–[38]. A more recent study evaluated the free energy values of the binding between the TCR and MHC-II in a multimeric complex (TCR-pMHC-II-CD4) anchored in a lipid membrane, but neglecting the energetic contribution of the peptide binding and the conformational changes linked to be simulated in aqueous solution or in a membrane environment [25]. Therefore, in this study, we developed a model in which MHC-II is anchored to a POPC membrane through part of its α and β heavy chains. Furthermore, we explored the differences in the conformational mobility and the energy components of MHC-II when bound to a small or large peptide, either as a water-soluble protein or anchored to a POPC membrane, in MD simulations.

### Equilibrium properties

**Figure 2:**
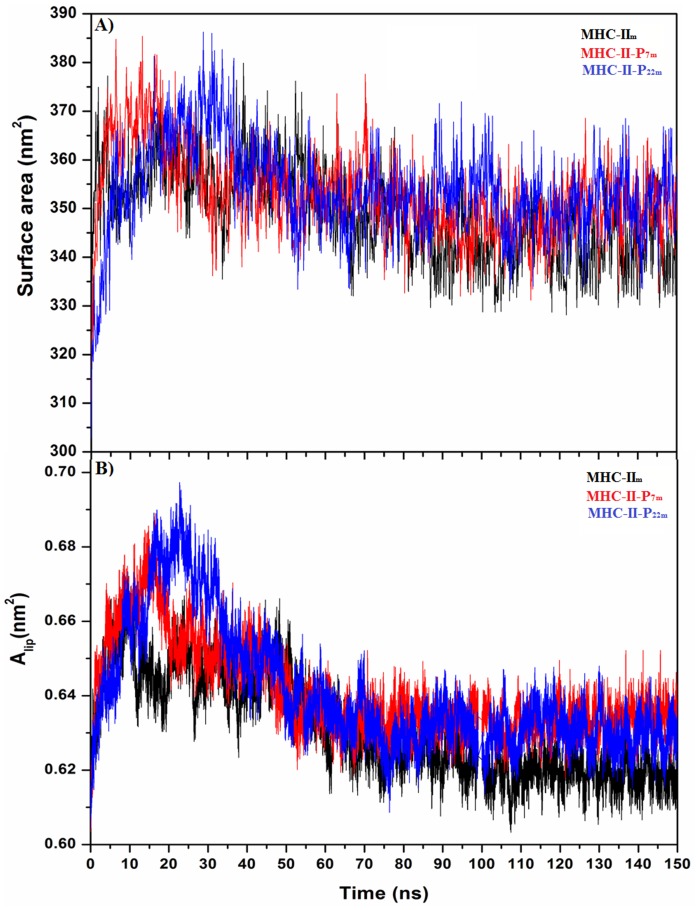
Membrane equilibrium after embedding MHC-II_m_ (black line), MHC-II-P_7m_ (red line), and MHC-II-P_22m_ (blue line) in a POPC membrane. The surface area (A) and (B) area per lipid (A_lip_) as a function of the simulation time show that both properties converged to stable values after 50 ns.

To determine when the POPC membrane with the embedded MHC-II molecule is in equilibrium, we analyzed the changes in several geometrical properties during the MD simulations time. Fig. 2 shows the time evolution of some properties in the bilayer environment, such as the area per lipid (A_lip_) and the total surface area for MHC-II_m_, MHC-II-P_7m_ and MHC-II-P_22m_. From figure 2, it is clear that A_lip_ was a slowly converging parameter, but reaching equilibrium over the 50 ns used in the MD simulations for the three systems. Furthermore, this A_lip_ value is in good agreement with those found for other protein-POPC-membrane systems [39]. The membrane surface area increased quickly in the first 20 ns, which could be due to a strong repulsive interaction among the lipid molecules and between the lipid and the MHC-II molecule, but these interactions reached an average value over the first 50 ns because of the conformational adjustment of the lipid and proteins, suggesting that the system is stable after this simulation time.

Several average geometrical properties, such as the α-carbon root-mean squared deviations (RMSD), intramolecular hydrogen bonds (HB_intra_), radius of gyration (R_g_), and apolar and polar solvent accessible surface areas (SASAs), were evaluated for MHC-II in its free and pMHC-II states in water and a lipid bilayer environment, however, for this latter case, only the soluble domain was considered to perform an appropriate comparison. Table 1 shows that most of the systems studied in aqueous solution converged within the first 15 ns, whereas the system modeled in the membrane environment reached equilibrium in a longer period of simulation time, over the first 30 ns, although this characteristic was more remarkable for MHC-II_m_, suggesting that the peptide coupled to the MHC-II molecule confers more stability to the MHC-II molecule in a membrane environment. Interestingly, the RMSD values were similar for the soluble and membrane-bound MHC-II (Table 1), revealing that the intrinsically mobility of MHC-II molecule is not affected by the environment. These MD simulations results indicate that both pMHC-II are more stable when the system includes a lipid membrane, however, in average their geometrical parameters took more time to reach convergence, but mainly for MHC-II_m_ (Table 1). A higher number of HB_intra_ was observed for the soluble and the membrane-bound pMHC-II complexes than for MHC-II_w_ and MHC-II_m_, pointing out that upon complex formation both bound states could be thermodynamically more stable. Although R_g_ and the apolar and polar SASAs did not show notable differences among most of systems, a clear increment in R_g_ and the apolar and polar SASA values is observed for MHC-II_m_ with respect to all the other systems. This result indicates that the lipid membrane environment confers an increase in the volume of MHC-II_m_, behavior that is not observed for MHC-II_m_ (Table 1). Furthermore, a decrease is experienced by MHC-II_m_ upon complex formation, result that is in agreement with experimental reports where an increase in the hydrodynamic radius for the peptide-free form of MHC-II (DRB1*0101) was observed with respect to pMHC-II [12], [13]. In summary, all these parameters indicate that the system did not undergo any significant conformational changes during the simulations.

**Table 1:** Geometrical properties of the peptide-free MHC-II and pMHC-II states in aqueous solution and pMHC-II anchored to a membrane.

System	HB_intra_	R_G_ (nm)	Apolar SASA (nm^2^)	Polar SASA (nm^2^)	Equilibrium RMSD (nm)
MHC-II_w_	258±9.0	2.37±0.02	111±2.1	113±2.0	0.54±0.05 (10 ns)*
MHC-II-P_7w_	268±10	2.44±0.03	115±2.7	114±2.6	0.51±0.06 (10 ns)*
MHC-II-P_22w_	287±10	2.41±0.02	116±2.0	114±2.4	0.41±0.02 (13 ns)*
MHC-II_m_	235±9.0	3.30±0.03	150±2.0	127±2.0	0.54±0.02 (30 ns)*
MHC-II-P_7m_	268±8.0	2.33±0.01	110±2.0	111±2.0	0.38±0.02 (15 ns)*
MHC-II-P_22m_	265±8.0	2.36±0.01	109±2.0	111±2.0	0.26±0.02 (20 ns)*

* Time at which the system had converged and the geometrical parameters were evaluated.

### Conformational mobility

**Figure 3:**
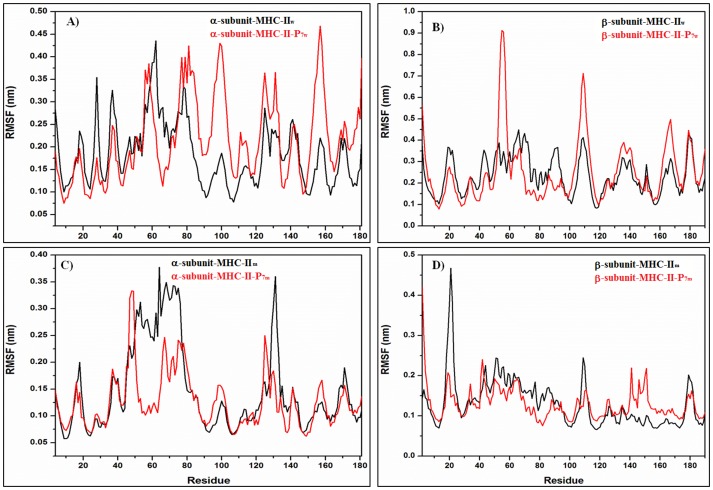
RMSF analysis of the water-soluble and membrane-bound MHC-II-P_7_ complex. A-B) The soluble peptide-free (MHC-II_w_, black line) and peptide-bound (MHC-II-P_7w_, red line) species. C-D) The membrane-bound peptide-free (MHC-II_m_, black line) and peptide-bound (MHC-II-P_7m,_ red line) complexes.

**Figure 4:**
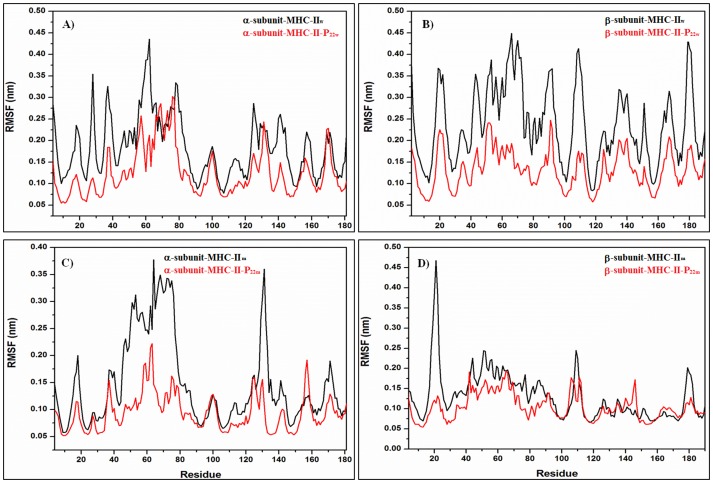
RMSF analysis of the soluble and membrane-bound MHC-II-P_22_ complex. A-B) The soluble peptide-free (MHC-II_w_, black line) and peptide-bound (MHC-II-P_22w,_ red line) complexes. C-D) The membrane-bound peptide-free (MHC-II_m_, black line) and peptide-bound (MHC-II-P_22m_, red line) complexes.

**Figure 5:**
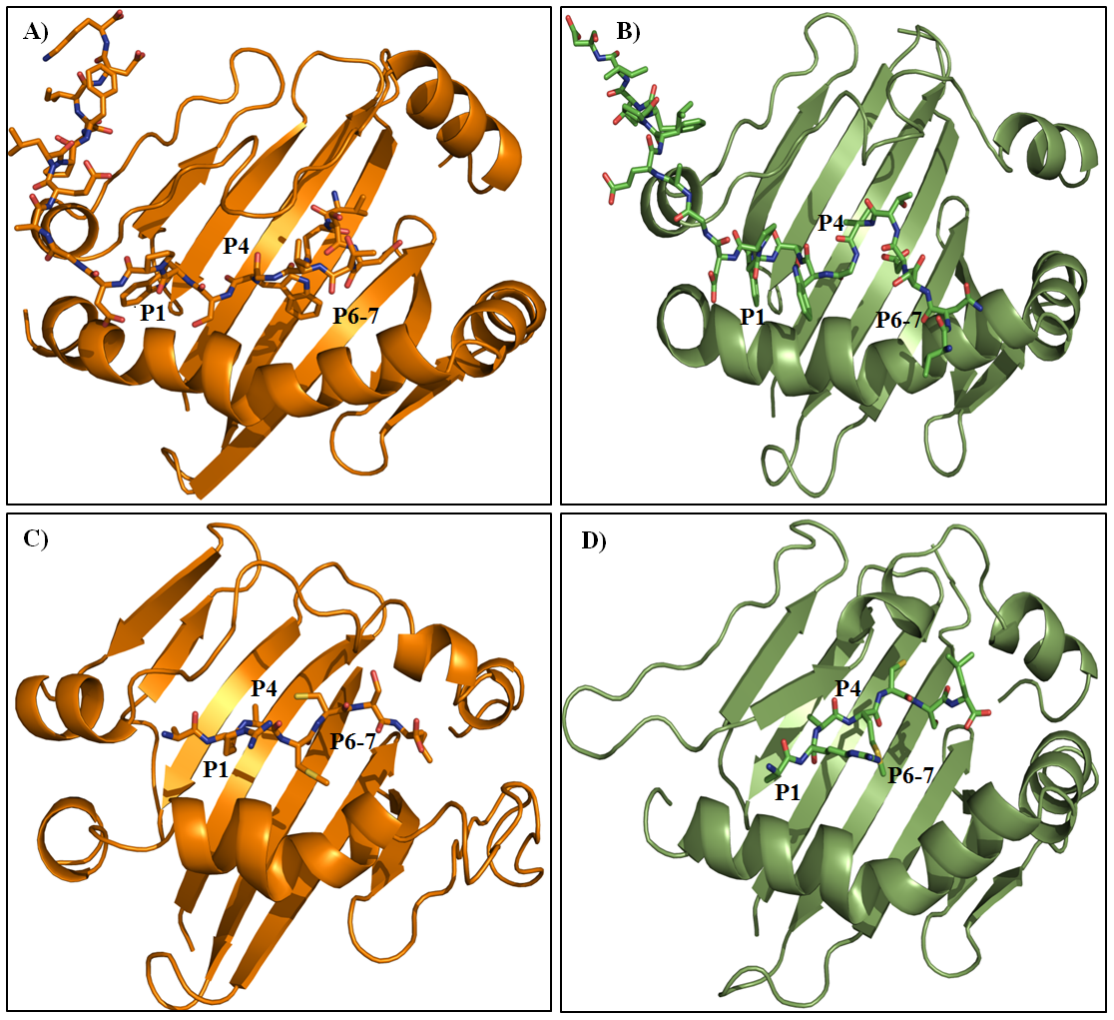
Average structures of the pMHC-II complexes. A) MHC-II-P_22w_, B) MHC-II-P_22m_, C) MHC-II-P_7w_ and D) MHC-II-P_7m_.

Root mean square fluctuations (RMSFs) over the α-carbon atoms were calculated using the last 85 and 100 ns of the MD simulation trajectory for the soluble and membrane systems, respectively, time at which both systems reached convergence (see Table 1). Fig. 3A and 3B show the RMSFs for MHC-II_w_ and MHC-II_m_ (black) and the MHC-II-P_7w_ and MHC-II-P_7m_ (red). These MD simulation results demonstrate that MHC-II-P_7w_ exhibited a reduction in conformational mobility for the first half of the α-subunit (4–70) but an increase for the other half (70–181) with respect to the conformational mobility of MHC-II_w_ (fig. 3A). The β-subunit exhibited an increase in its fluctuations for MHC-II-P_7w_ relative to MHC-II_w_ in several protein regions (fig. 3B) but was most remarkable for the regions (50–60) and (100–120). Fig. 3C and 3D show the RMSFs for the MHC-II_m_ (black) and MHC-II-P_7m_ (red), from which can be noted that there was a significant decrease for two regions localized in the α-subunit (residues 50–80 and 125–140), whereas for the β-subunit a remarkable decrease in region (15–30) was observed for MHC-II-P_7m_ with respect to MHC-II_m_. Fig. 4 depicts the RMSFs for MHC-II_w_ and MHC-II_m_ (black) and MHC-II-P_22w_ and MHC-II-P_22m_ (red). Fig. 4A and B show that MHC-II-P_22w_ experienced a significant decrease in conformational mobility in both of its subunits with respect to MHC-II_w_, and this behavior was more remarkable for the β-subunit (fig. 4B). In MHC-II-P_22m_, both of its subunits also experienced a decrease in mobility with respect to MHC-II_m_, but was more remarkable for two protein regions in α-subunit (residues 35–85 and 110–140) and one in β-subunit (residues 15–30) (fig. 4D). Interestingly, the structural mobilities of the MHC-II-P_7m_ and the MHC-II-P_22m_ with respect to the MHC-II_m_ were similar (fig. 3C-D and 4C-D), and the protein regions that were mainly affected in both complexes were those located in the α and β light chains of the peptide-binding groove. For all the complexes, it was noticed a loss of α-helical structure in the peptide-binding groove because of the interactions with the peptides (fig. 5), however, for the case of MHC-II-P_7m_, a β-sheet structure is formed (fig. 5D).

For the soluble species, there was an increase in conformational mobility for the MHC-II-P_7w_ complex and a reduction in conformational flexibility for the complex with the larger peptide (MHC-II-P_22w_), together with structural changes observed even in protein regions that are far from the peptide-binding groove (6A and 6E). These results indicate that all the systems modeled as soluble molecules exhibited more flexible behavior than those anchored to a bilayer membrane.

**Figure 6:**
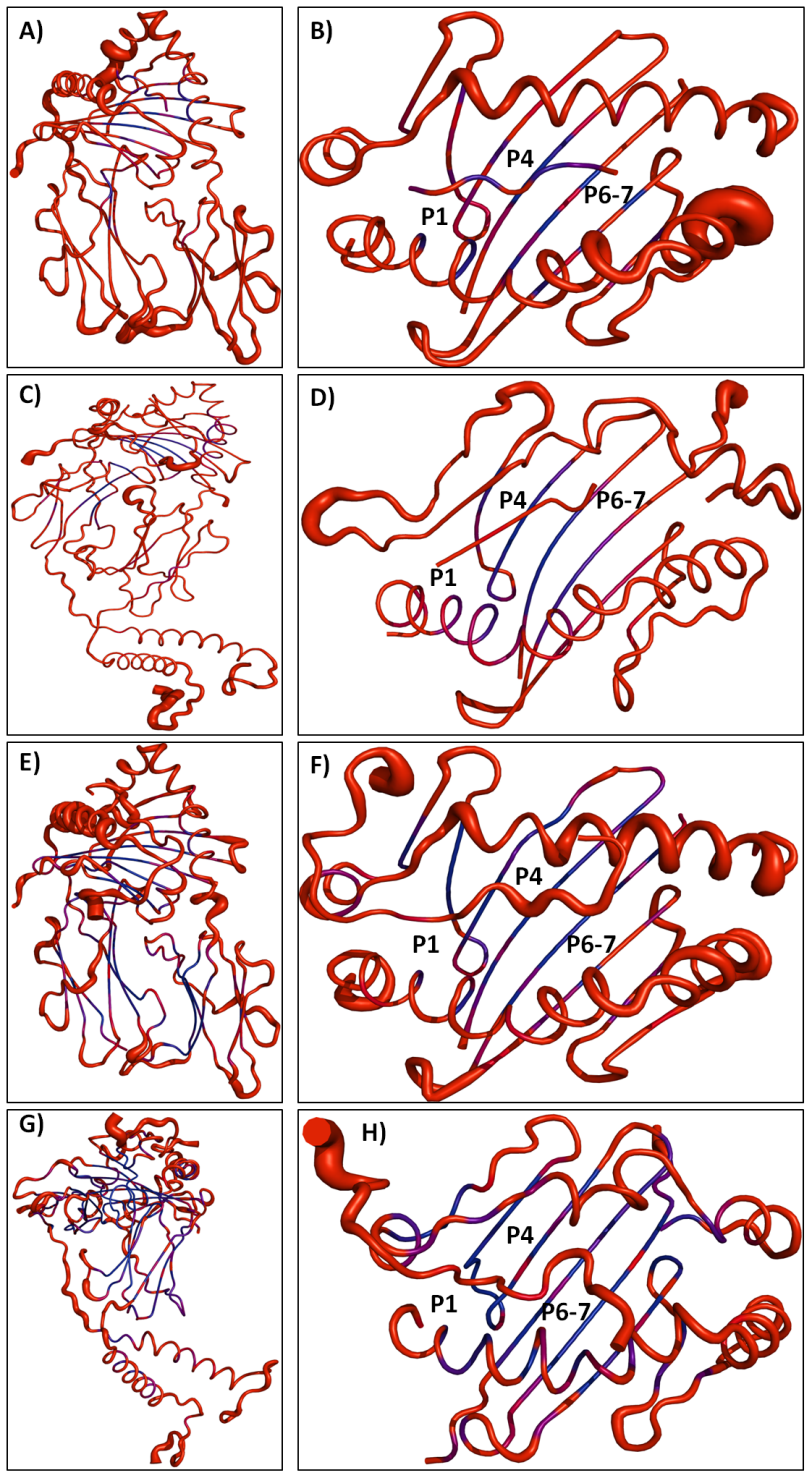
pMHC-II complexes color-coded according to their B-factors. A-B) MHC-II-P_7w_ complex. C-D) MHC-II-P_7m_. E-F) MHC-II-P_22w_ and G-H) MHC-II-P_22m_. Complexes are drawn in cartoon representation and color-coded according to the B-factor of Cα atom, from blue (lowest B factor: less than 30 Å^2^) to red (highest B factor: greater than 50 Å^2^). B-factors were obtained from the average RMSF values.

To explore and compare the flexibility properties of soluble peptides (P_7w_ and P_22w_) and membrane-bound (P_7m_ and P_22m_) coupled to MHC-II in both simulation conditions, the b-factor of α-carbon was evaluated during the equilibrium time for both peptides. Figure 6 presents the conformational behavior of P_7_ and P_22_ into the peptide binding site for the soluble (fig. 6B and 6F) and membrane-bound (fig. 6D and 6H) complexes. From this figure, it can be seen that P_7w_ and P_7m_ underwent almost the same conformational flexibility into the peptide binding groove, whereas that P_22m_ is clearly more flexible than P_22w_, above all in the C-terminal region, site that is mainly stabilized by residues beyond the peptide binding groove (fig. 6H).

### Structural analysis of the peptide-bound complex

**Figure 7:**
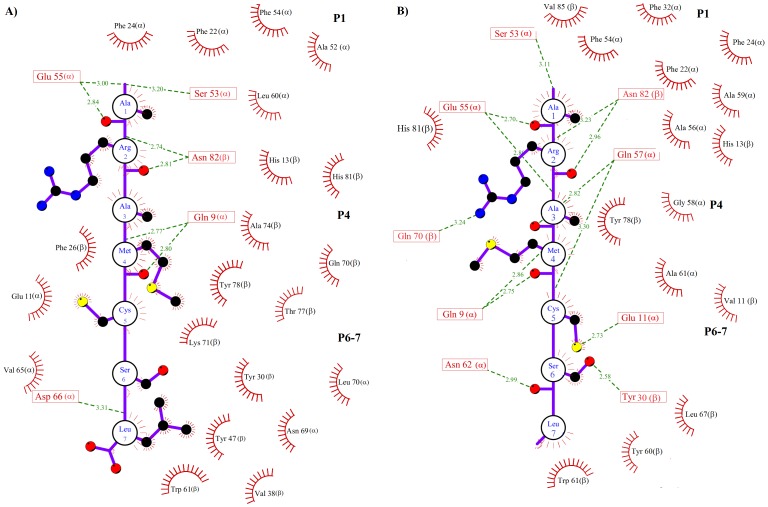
Schematic MHC-II-P_7_ representation. A) Map of the interactions that stabilize the soluble MHC-II-P_7w_ complex. B) Map of the interactions that stabilize the membrane-bound MHC-II-P_7m_. The residues of P_7_ are represented by a single circle. Only the side chains of P_7_ involved in hydrogen bonds or hydrophobic contacts are shown explicitly. MHC-II residues participating in hydrogen bonds (green dotted lines) are represented by a single box, and hydrophobic contacts are represented by red half circles.

**Figure 8:**
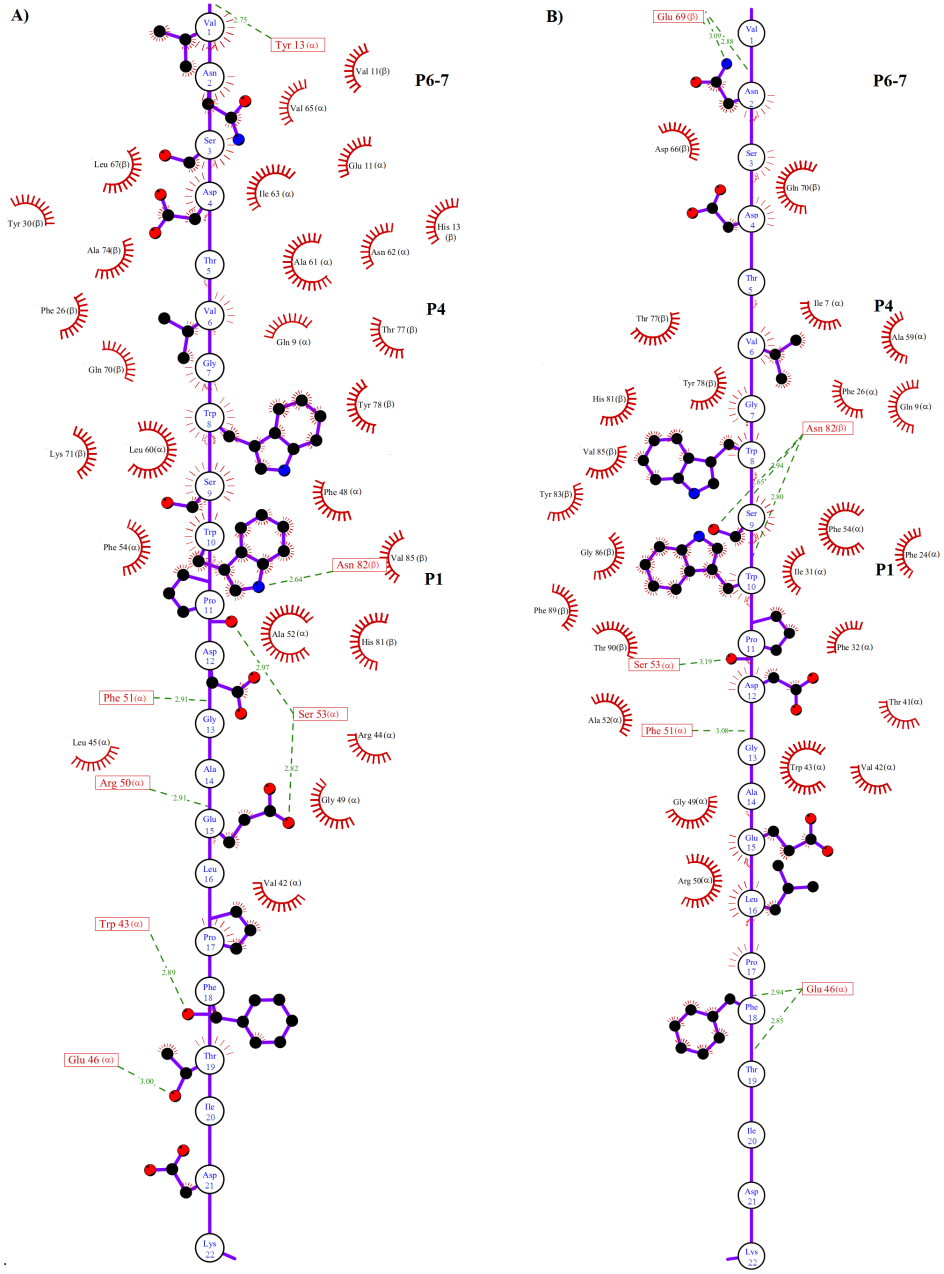
Schematic representation of the non-covalent interactions between MHC-II and P_22_. A) Map of the interactions that stabilize the soluble MHC-II-P_22-w_ complex. B) Map of the interactions that stabilize the membrane-bound MHC-II-P_22m_. The residues of P_22_ are represented by a single circle. Only the side chains of P_22_ involved in hydrogen bonds or hydrophobic contacts are shown explicitly. MHC-II residues participating in hydrogen bonds (green dotted lines) are represented by a single box, and hydrophobic contacts are represented by red half circles.

It has been stated that several pockets (P1-P11) are important for epitope-MHC-II recognition [16], with the P1 pocket being particularly important [13], [58]. This pocket is formed by bulky hydrophobic residues such as Trp, Tyr, Phe, Leu and Ile [38]. The previously reported importance of P1 is in accordance with our docking and MD simulations, in which both epitopes interacted with P1, P4 and P6-7 (fig. 5), but is slightly in conflict with the results of our previous study, in which the epitope was docked using a focused docking approach and interacted with only P4, P6-7 and P9 [26]. Our analysis in this study indicates that both peptides reached a tight and long-lived conformation in the peptide-binding groove within the first 5 ns. These interactions were stabilized by residues in P1, P4 and P6-7 (fig. 7–8).

To further explore the detailed map of the interactions, the average structures were calculated for MHC-II-P_7w_, MHC-II-P_7m_, MHC-II-P_22w_ and MHC-II-P_22m_ through the MD simulations time period, during which the systems reached equilibrium (see Table 1). Fig. 5 shows the average structures of the soluble and membrane-bound complexes formed between P_7-22w_ (fig. 5A and 5C) or P_7-22m_ (fig. 5B and 5D) and MHC-II. In both cases, the peptide was bound in the peptide-binding groove of MHC-II in an extended polyproline type II-like conformation (fig. 5), as observed for other MHC-II-peptide complexes [14], [16], [57]. Furthermore, several residues reported to be important for stabilizing MHC-II-peptide complexes [13], [59]–[61] were also found in our pMHC-II complexes (highlighted in red, Table 2). In the case of MHC-II-P_7w_ and MHC-II-P_7m_, it was observed that P_7_ was totally covered by MHC-II (fig. 5C and 5D), whereas for P_22_, only 16 residues are buried in the peptide-binding groove (fig. 5A and 5B). However, despite these features, both peptides were stabilized by residues located in P1, P4 and P6-7, and most of these interactions were hydrophobic in nature (fig. 7 and 8). Hydrogen bonds, to a lesser degree, also seem to be crucial in maintaining these complexes. In the case of MHC-II-P_7w_, it was observed that the MHC-II-P_7w_ complex had a greater number of hydrophobic interactions than the MHC-II-P_7m_ (fig. 7), whereas that MHC-II-P_7m_ showed a greater number of hydrogen bonds. Comparisons of the average structures obtained through MD simulations revealed that P_7_ in MHC-II-P_7w_ is bound by almost the same residues in P1, P4 and P6-7 as observed in the crystallographic complex (1D5 M); however, a greater number of hydrogen bonds are present in the latter complex because of the chemical modifications of the peptide mimetic species that are not present in P_7w_ (data not shown). In the case of MHC-II-P_22w_ and MHC-II-P_22m_, P_22_ underwent for both situations approximately the same number of hydrophobic interactions and hydrogen bonds (fig. 8). Nonetheless, unlike the MHC-II-P_7w_ and MHC-II-P_7m_ complexes, in which all the residues that make up the peptide were stabilized by residues lining the peptide-binding groove, the MHC-II-P_22w_ and MHC-II-P_22m_ had a group of residues (Leu16 to Lys22) that were stabilized by residues outside of the peptide-binding groove (fig. 5A and 5B). Furthermore, it is worth noting that as observed for the MHC-II-P_7m_ complex, P_22_ in the MHC-II-P_22m_ complex was primarily bound by residues in P1 and P4 (Table 2). The C-terminal region in MHC-II-P_22w_ was more rigid than that of MHC-II-P_22m_ as a result of the higher number of non-covalent interaction between P_22w_ and residues beyond the peptide binding site (fig. 6H and 8A). Thus, these results indicate that the membrane influence the peptide recognition process, suggesting that as this system is found biologically anchored to a lipid membrane, it would be better to use the whole system instead MHC-II without membrane to obtain insight into the immunogenic properties of peptides.

**Table 2:** pMHC-II interactions between peptide residues and pockets (Ps).

Pocket	MHC-II peptide-binding groove residues
	**MHC-II-P_22w_**
P1	αPhe48, αAla52, **αPhe54**, βHis81, βVal85 and HB with αPhe51, αSer53, βAsn82.
P4	αGln9, αLeu60, βPhe26, **βGln70, βLys71**, βThr77and **βTyr78**.
P6-7	αGlu11, βVal11, **αAla61, αAsn62,** αIle63, αVal65, βHis13, βTyr30, βLeu67, βAla74 and HB with αTyr13.
	**MHC-II-P_22m_**
P1	αPhe24, αPhe32, αAla52, **αPhe54**, βHis81, βTyr83, βVal85, βGly86, βPhe89, βThr90, αIle31 and HB with αPhe51, αSer53, βAsn82.
P4	αIle7, αGln9, αAla59, βPhe26, βThr77and **βTyr78**.
P6-7	βAsp66, βGln70 and HB with βGlu69.
	**MHC-II-P_7w_**
P1	αPhe22, αPhe24, αAla52, **αPhe54**, αLeu60, βHis13, βHis81 and HB with αSer53, αGlu55, βAsn82.
P4	αGlu11, βPhe26, βGln70, **βLys71,** βAla74, **βThr77, βTyr78** and HB with αGln9.
P6-7	αVal65, αAsn69, αLeu70, βTrp61, βTyr30, βVal38, βTyr47 and HB with αAsp66.
	**MHC-II-P_7m_**
P1	αPhe22, αPhe24, αPhe32, **αPhe54**, αAla56, αAla59, βHis13, βHis81, βVal85 and HB with αSer53, αGlu55, βAsn82.
P4	αGly58, αAla61, βVal11, **βTyr78**, and HB with αGln9, αGlu11 and βGln70.
P6-7	βTyr60, βTrp61, βLeu67 and HB with αAsn62 and βTyr30.

Residues reported to be important for stabilizing MHC-II-peptide complexes [13], [59]–[61] are highlighted in bold.

### Thermodynamic properties

To determine the energetic values that drive pMHC-II complex stabilization, two types of short-range energies, Lennard-Jones (LJ-SR) and Coulomb (Coul-SR) energies, were considered for the water-soluble systems and the membrane-bound pMHC-II complexes, together with the calculation of the intrinsic energy for each molecule involved in complex formation. Table 3 shows that the Coul-SR energies have comparable values for the complex between MHC-II and P_7_, both in water (MHC-II-P_7w_) and in a membrane-embedded environment (MHC-II-P_7m_), whereas that a slightly higher Lennard-Jones energy value was observed for MHC-II-P_7w_. On the other hand, more favorable LJ-SR and Coul-SR components were observed for MHC-II-P_22m_ relative to MHC-II-P_22w_, with more energetic Coul-SR and LJ-SR values of -31 kJ and -19 kJ, respectively. This result is at odds with that observed for the MHC-P_7w_ and MHC-II-P_7m_, for which a more favorable LJ-SR energy value of -27 kJ was observed for MHC-II-P_7w_ (Table 3). However, for all the complexes, the total energy interaction was dominated by the Coul-SR energies.

**Table 3:** Free energy values for the interactions in the MHC-II complexes.

System	Coul-SR (kJ)	LJ-SR (kJ)
**MHC-II-P_7w_**	**−370±20**	**−214±5.0**
**MHC-II-P_7m_**	**−370±22**	**−187±5.0**
**MHC-II-P_22w_**	**−443±21**	**−371±11**
**MHC-II-P_22m_**	**−474±16**	**−390±6.0**

In contrast, the comparison of the intrinsic energies for each of the components involved in stabilizing the complexes showed that both the LJ-SR and Coul-SR energies were more favorable when MHC-II was membrane anchored and forming a complex (MHC-II-P_7m_ and MHC-II-P_22m_) than the soluble species (MHC-II_w_, MHC-II-P_7w_ and MHC-II-P_22w_). For the membrane-bound species, a more energetic LJ-SR component was observed for MHC-II in the complex forms (MHC-II-P_7m_, MHC-II-P_22m_) than in its free state (MHC-II_m_), whereas that the opposite was observed with respect to the Coul-SR energies. However, both energy components were much more favorable for the membrane-bound systems than for the soluble species, indicating that MHC-II becomes more energetically stabilized in a membrane-bound environment.

The comparison of P_7_ and P_22_ free in solution or bound to a soluble or membrane-bound MHC-II molecule revealed a more favorable Coul-SR value for the membrane-bound situation. In fact, this energy component was almost the twice those observed for the soluble complex and even that of the free peptide in solution (Table 4). There were only small changes in the LJ-SR energies; however, it is worth mentioning that this energy component exhibited a more favorable energy for the complex free in solution (Table 4), a result that is most likely due to the greater energy of the LJ-SR interactions in its own structure than those in the almost linear conformation of the peptide in the peptide-binding groove. Overall, these results indicate enhanced peptide and MHC-II stability in the membrane-bound complex than in the free complex in water.

**Table 4:** Intrinsic energies for each component of the MHC-II complexes.

System	Coul-SR (kJ)	LJ-SR (kJ)
**MHC-II_w_** a	**−13620±45**	**−8830±16**
**MHC-II_m_** a	**−29625±87**	**−11917±33**
**MHC-II-P_7w_** a	**−14500±77**	**−9420±43**
**MHC-II-P_7m_** a	**−28757±26**	**−12070±16**
**MHC-II-P_22w_** a	**−15020±63**	**−9610±14**
**MHC-II-P_22m_** a	**−28360±88**	**−12143±7.0**
**P_7w_** b	**−120±3.0**	**−60±1.0**
**P_7m_** c	**−250±5.0**	**−68±1.0**
**P_7_** d	**−140±0.6**	**−70±0.4**
**P_22w_** b	**−570±10**	**−260±3.3**
**P_22m_** c	**−1077±15**	**−277±5.0**
**P_22_** d	**−740±10**	**−380±10**

a Intrinsic energies of MHC-II for each system.

b Intrinsic energies of P_7w_ and P_22w_ when in the MHC-II-P_7w_ and MHC-II-P_22w_ complexes, respectively.

c Intrinsic energies of P_7m_ and P_22m_ when in MHC-II-P_7m_ and MHC-II-P_22m_, respectively.

d Intrinsic energies of P_7_ and P_22_ when free in solution.

### Conclusion

Previous experimental studies have revealed that peptide-free MHC-II and pMHC-II complexes have different physical properties, for example, different hydrodynamic radii and thermal stabilities [15]. Computational studies comparing pMHC-II_w_ to the isolated peptide-binding groove domain in complex with a peptide have demonstrated that neglecting some domains has an important influence on conformational behavior and the energetics of complex formation [23], which are crucial to make a correct determination of the epitope binding efficiency. These parameters are crucial because it is well known that only peptides participating in high-affinity interactions with MHC molecules are recognized as T-cell epitopes [62]. A more appropriate study has been recently conducted using MD simulation procedures in which the TCR-pMHC-II-CD4 complex was simulated in a membrane environment [25]; however, that study did not explore the differences in conformational behavior or energetic components between systems simulated as water-soluble molecules and those simulated as complexes in a membrane environment.

Furthermore, it has been reported that the understanding of the structure, stability and longevity of pMHC complexes in the membrane lipids is of general interest because of the importance of these properties in regulating differential T lymphocyte activation [35]. Therefore, our goal in this work was to gain insight into the structural and energetic changes that take place for peptide-free MHC-II and pMHC-II complexes in water and membrane environments. We also investigated the structural and energetic changes when these complexes were anchored to a lipid membrane by using a more realistic molecular simulation model as that proposed to simulate cytochrome P450 2C9 [63].

The conformational mobility analysis showed distinct behaviors for the water-soluble and membrane-bound forms. The soluble complexes underwent distinct conformational changes that depended on the peptide size, and there were conformational changes far from the peptide-binding groove. For the membrane-bound complexes, both complexes exhibited reduced conformational mobility in the region restricted to the peptide-binding groove. Furthermore, intramolecular energy analysis indicated that the components in the membrane-bound complexes were more energetically favored, whereas the intermolecular energy analysis of MHC-II bound to P_7_ or P_22_ yielded similar values for the soluble and membrane-bound MHC-II-P_7_ complexes, but more favorable for MHC-II-P_22m_. Interestingly, despite the differences in epitope size and MD simulation environments, both peptides were stabilized primarily by residues lining P1, P4, P6-7. However, some discrepancies were observed in the conformational mobility of P_7_ and P_22_ in the membrane-bound complexes. These discrepancies were attributed to differences in the map of interactions between the residues lining P1, P4, P6-7 and both peptides (fig 7 and 8). Therefore, these results suggest that simulating the system in a membrane environment yield some differences in the map of the interactions between the epitope and other MHC-II molecules and could provide more suitable information about the changes in energy and conformational mobility upon complex formation. Although the methodology used here to simulate a membrane environment took more time to reach convergence than for the soluble system, our methodology could be good enough to obtain the energetic behavior for a list of epitopes because, according to our results, complex formation does not involve significant conformational changes. Therefore, the entropic component would play a minor role. However, the calculation of the relative free energy for a soluble MHC-II-peptide complex using methods such as the molecular mechanics generalized Born surface area (MM/GBSA) [64] must be used very carefully because although this method allow to estimate relative binding free energies, the final binding free energy value is estimated by subtracting the entropic component, which in the case of soluble MHC-II-peptide complexes would result in the overestimation of the values relative to those actually experienced in a membrane-bound environment.
